# Development of Detection Antibody Targeting the Linear Epitope in SARS-CoV-2 Nucleocapsid Protein with Ultra-High Sensitivity

**DOI:** 10.3390/ijms25084436

**Published:** 2024-04-18

**Authors:** Feng Wu, Yike Jiang, Hongtian Yang, Lan Ma

**Affiliations:** 1 Institute of Biopharmaceutical and Health Engineering, Tsinghua Shenzhen International Graduate School, Tsinghua University, Shenzhen 518055, China; wf19@tsinghua.org.cn (F.W.); yanght23@mails.tsinghua.edu.cn (H.Y.); 2 Shenzhen Institute of Drug Control, Shenzhen 518057, China; 3 Institute of Biomedical Health Technology and Engineering, Shenzhen Bay Laboratory, Shenzhen 518132, China; jiangyk@szbl.ac.cn; 4 State Key Laboratory of Chemical Oncogenomics, Tsinghua Shenzhen International Graduate School, Tsinghua University, Shenzhen 518055, China

**Keywords:** SARS-CoV-2, N protein, antibody, linear epitope, conserved sequence

## Abstract

The COVID-19 pandemic caused by SARS-CoV-2 highlighted the importance of reliable detection methods for disease control and surveillance. Optimizing detection antibodies by rational screening antigens would improve the sensitivity and specificity of antibody-based detection methods such as colloidal gold immunochromatography. In this study, we screened three peptide antigens with conserved sequences in the N protein of SARS-CoV-2 using bioinformatical and structural biological analyses. Antibodies that specifically recognize these peptides were prepared. The epitope of the peptide that had the highest binding affinity with its antibody was located on the surface of the N protein, which was favorable for antibody binding. Using the optimal antibody that can recognize this epitope, we developed colloidal gold immunochromatography, which can detect the N protein at 10 pg/mL. Importantly, this antibody could effectively recognize both the natural peptide antigen and mutated peptide antigen in the N protein, showing the feasibility of being applied in the large-scale population testing of SARS-CoV-2. Our study provides a platform with reference significance for the rational screening of detection antibodies with high sensitivity, specificity, and reliability for SARS-CoV-2 and other pathogens.

## 1. Introduction

The coronavirus disease 2019 (COVID-19) is caused by the severe acute respiratory syndrome coronavirus 2 (SARS-CoV-2) [1]. According to the World Health Organization (WHO), more than 770 million COVID-19 cases have been reported and more than 6.9 million people have died from COVID-19 worldwide.

SARS-CoV-2 is a single-stranded RNA virus [2]. The structural proteins of SARS-CoV-2 are composed of nucleocapsid (N), membrane (M), envelope (E), and spike (S) proteins [3]. The N protein of SARS-CoV-2 is relatively stable and conserved [4,5]. Therefore, the N protein is often selected as the target of detecting SARS-CoV-2 [6,7,8,9,10]. The N protein is also used as the therapeutic target of SARS-CoV-2. N protein-targeting drugs including an antibody and some small molecules have been developed [11,12,13,14]. The N protein may also serve as a potential target for vaccine development [2,14].

SARS-CoV-2 is still a highly transmissible pathogen nowadays and it continues to evolve [15]. Rapid, accurate, and reliable detection of SARS-CoV-2 is critical in preventing the transmission of SARS-CoV-2. Nucleic acid tests and lateral flow immunoassays (LFIAs) are the mainstays of detecting SARS-CoV-2. Nucleic acid tests (e.g., RT-PCR) provide accurate and sensitive detections. Performing these tests, however, often takes a long time, and commercial RT-PCR systems usually take hours to complete these tests [16]. Nucleic acid tests also require specialized instruments and trained personnel. In contrast, LFIAs (e.g., colloidal gold immunochromatography) provide fast and easy-to-operate detections at a relatively low cost [10], which is advantageous in the home-based use and large-scale population testing of SARS-CoV-2, particularly in underdeveloped areas. In addition to nucleic acid tests and LFIAs, multiple PCR-free detection strategies such as using electrochemical and electrical sensors have been developed to detect SARS-CoV-2 [17].

The sensitivity and specificity of LFIAs depend highly on the characteristics of antibodies, which can be greatly influenced by the type of antigen. Using high-molecular-weight recombinant proteins or pathogens such as inactivated viruses that have multiple epitopes as antigens could produce antibodies that recognize several conformational and/or linear epitopes. These antibodies bind to epitopes randomly. In contrast, using linear peptides with conserved sequences as antigens could produce antibodies that recognize a single conserved epitope with high specificity and sensitivity, avoiding potential interferences from mutations. Therefore, these antibodies are suitable for application in detection across variants. Importantly, antibodies that bind to a specific peptide can be obtained by screening the dominant linear epitopes with bioinformatical and structural biological methods when the target is clear. This solves the long-standing problem that detection antibodies can only be prepared with random screening, but not with precise design.

**Figure 1:**
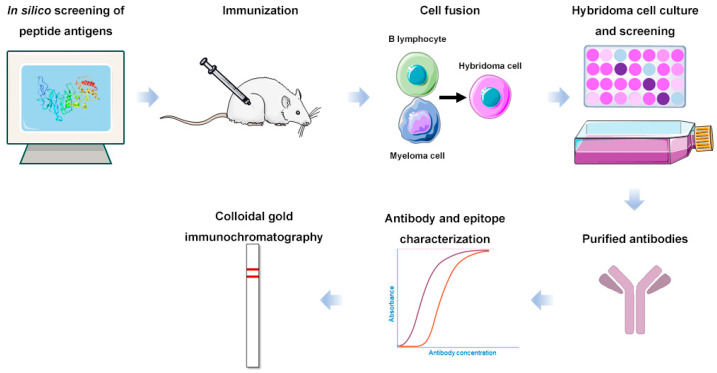
Scheme of the overall process of this research.

In this study, we rationally screened linear peptide antigens with conserved sequences in the N protein of SARS-CoV-2 using bioinformatical and structural biological analyses. We prepared monoclonal antibodies (mAbs) that specifically recognize these antigens using hybridoma technology, aiming to develop a sensitive LFIA for SARS-CoV-2 detection (Figure 1). We clarified the epitope of the optimal linear peptide and prepared colloidal gold immunochromatographic strips with the antibody targeting this epitope, which demonstrated an ultra-high sensitivity for the N protein of SARS-CoV-2.

## 2. Results

### 2.1. Preparation of Antibodies Targeting Peptide Antigens with Conserved Sequences

**Figure 2:**
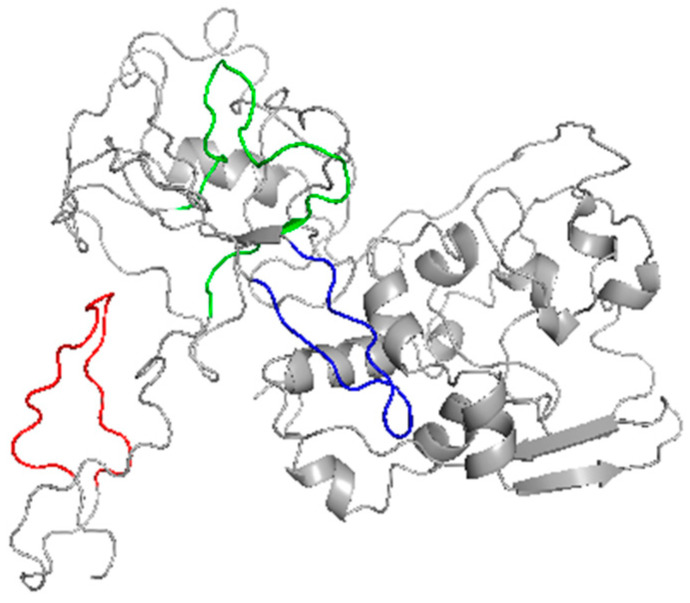
The structures of the linear peptides in the N protein of SARS-CoV-2. CoV-NP1, CoV-NP2, and CoV-NP3 are labeled in red, green, and blue, respectively.

We identified three linear peptides (CoV-NP1, CoV-NP2, and CoV-NP3) with conserved sequences in the N protein as the antigens of SARS-CoV-2. The structures of these peptides in the N protein are shown in Figure 2.

Immunizing mice with these peptide antigens followed by hybridoma screening yielded four positive clones for CoV-NP1, three positive clones for CoV-NP2, and two positive clones for CoV-NP3 that could recognize the N protein of SARS-CoV-2.

**Figure 3:**
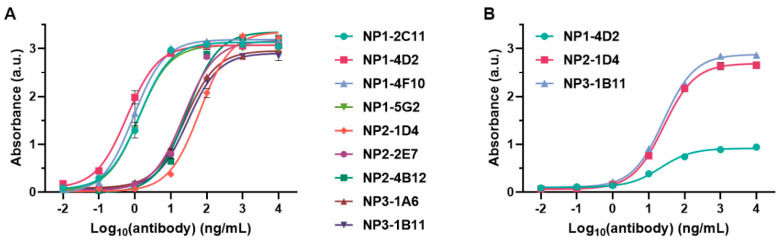
Binding of antibodies to (**A**) recombinant N protein and (**B**) peptide antigens.

We investigated the binding of antibodies produced by these positive clones to the recombinant N protein. As shown in Figure 3A, four antibodies (2C11, 4D2, 4F10, and 5G2) that were specific to CoV-NP1 had the highest affinities for the N protein. The EC_50_ values were 1.41, 0.71, 1, and 1.08 ng/mL, with antibody 4D2 having the smallest EC_50_ value. Three antibodies (1D4, 2E7, and 4B12) that were specific to CoV-NP2 had lower affinities for the N protein, with EC_50_ values of 60.93, 20.36, and 26.64 ng/mL, respectively. Two antibodies (1A6 and 1B11) that were specific to CoV-NP3 had similar affinities to 2E7 and 4B12 for the N protein, with the EC_50_ values being 25.51 and 29.66 ng/mL, respectively.

We also characterized the binding of 4D2, 1D4, and 1B11 to their corresponding peptide antigens (Figure 3B), where the EC_50_ values were 23.1, 26.8, and 24.8 ng/mL, respectively, suggesting that these antibodies had similar affinities with their peptide antigens. The low absorbance of 4D2 binding to CoV-NP1 was because of the low coating efficiency of CoV-NP1 on the ELISA plate.

### 2.2. Investigation of the Epitope in CoV-NP1

**Figure 4:**
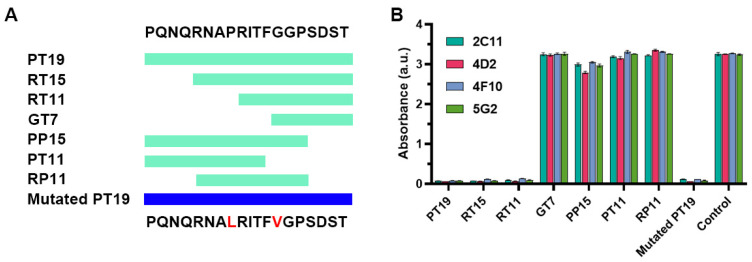
Identification of the epitope that can be recognized by CoV-NP1-targeting antibodies. (**A**) Sequences of truncated peptides for studying the epitope and sequence of the mutated CoV-NP1. The light green and blue bands indicate the coverage of the sequences. Mutations are labeled in red. (**B**) Competition between different peptides and the N protein for binding with CoV-NP1-targeting antibodies. PBS was used as the control.

**Figure 5:**
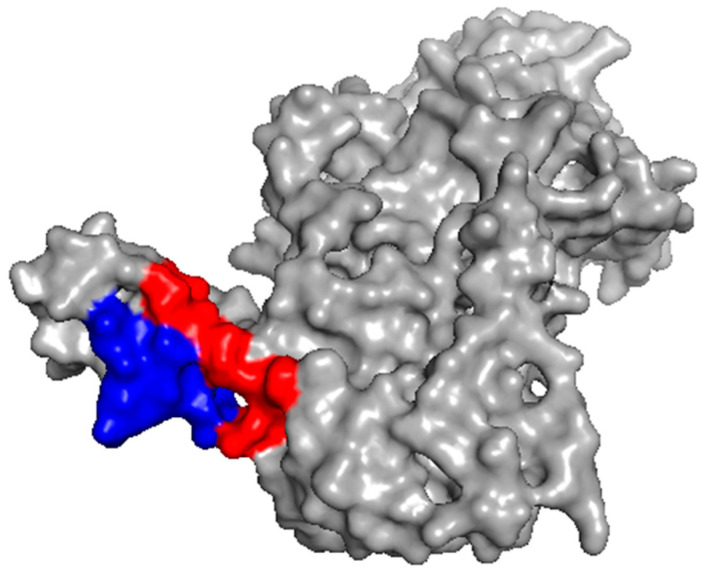
Location of the epitope in the N protein of SARS-CoV-2. The red region is the linear epitope RITFGGPSDST. The blue region is the rest residues of peptide PT19 (i.e., PQNQRNAP).

We investigated the epitope that could be recognized by the CoV-NP1-targeting antibodies, which had high affinities with the N protein. Based on the original sequence of CoV-NP1 PQNQRNAPRITFGGPSDST (PT19), we designed six truncated peptides (i.e., RNAPRITFGGPSDST (RT15), RITFGGPSDST (RT11), GGPSDST (GT7), PQNQRNAPRITFGGP (PP15), PQNQRNAPRIT (PT11), and RNAPRITFGGP (RP11)) (Figure 4A). We used these six truncated peptides together with PT19 (CoV-NP1) to compete with the N protein for binding with the CoV-NP1-targeting antibodies. A peptide that binds strongly with the antibody can inhibit the binding between the N protein and the antibody. The competition results shown in Figure 4B indicate that the antibodies strongly bonded with peptides PT19, RT15, and RT11. The shared sequence of these three peptides was RITFGGPSDST, meaning that this 11-mer peptide was the epitope of CoV-NP1. This peptide is located on the surface of the N protein (Figure 5), which is favorable for the binding of the Fab fragment of the antibodies. According to Peng et al., peptide RITFGGPSDST is located at the disordered region of the N protein [18]. Its binding with the antibody was therefore less influenced by steric hindrance, which also accounted for the high binding affinity between the CoV-NP1-targeting antibodies and the N protein (Figure 3A).

A recently published work reported four dominant epitopes in the N protein of SARS-CoV-2 using HLA peptidomics [19]. One of these epitopes, APRITFGGP, had a similar sequence to our epitope RITFGGPSDST, suggesting that our epitope is a dominant linear epitope in the N protein that can produce antibodies with a higher affinity than other epitopes.

### 2.3. Recognition of Mutated Peptide Antigen by CoV-NP1-Targeting Antibodies

Although the sequence of CoV-NP1 was conserved, mutation P13L or G18V did occur. We prepared a mutated peptide containing these two mutations (i.e., mutated PT19 with the sequence of PQNQRNALRITFVGPSDST) (Figure 4A). The competition results indicated that all four CoV-NP1-targeting antibodies still effectively recognized this mutated peptide (Figure 4B).

**Figure 6:**
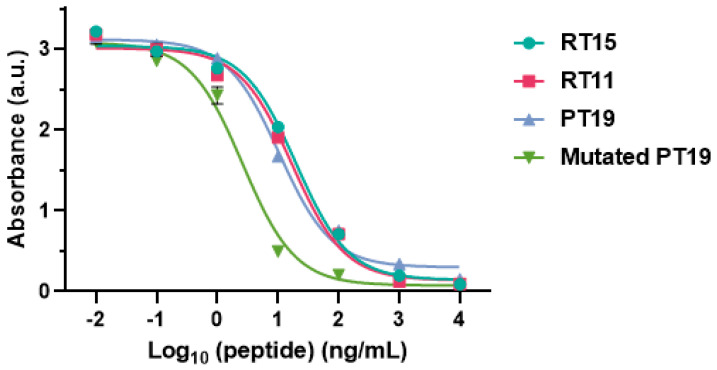
Inhibition of the binding between 4D2 and the N protein by different peptides.

We further quantitatively characterized the abilities of the original peptide PT19, the truncated peptides RT15 and RT11, and the mutated PT19 on suppressing the binding between CoV-NP1-targeting antibody 4D2 and the N protein (Figure 6). The IC_50_ values for RT15, RT11, PT19, and mutated PT19 were 19.1 ng/mL, 16.6 ng/mL, 10.2 ng/mL, and 2.1 ng/mL, respectively.

The IC_50_ value for mutated PT19 was the lowest, suggesting that antibody 4D2 had the strongest binding with mutated PT19. The proline (P)-to-leucine (L) mutation in PT19 occurred outside the sequence of the epitope, and thus had a negligible effect on the binding with antibody 4D2. The valine (V)-to-glycine (G) mutation in the peptide also did not affect its binding with the antibody even though it occurred within the epitope sequence since this was a non-disruptive mutation. Glycine has similar physicochemical properties to valine. They are nonpolar hydrophobic amino acids with similar structures. Hence, this mutation does not affect the recognition by the antibody. Additionally, two methyl groups on valine may increase the contact area between the antibody and antigen and thus strengthen their binding.

### 2.4. Detection of the SARS-CoV-2 N Protein Using Colloidal Gold Immunochromatography

**Figure 7:**
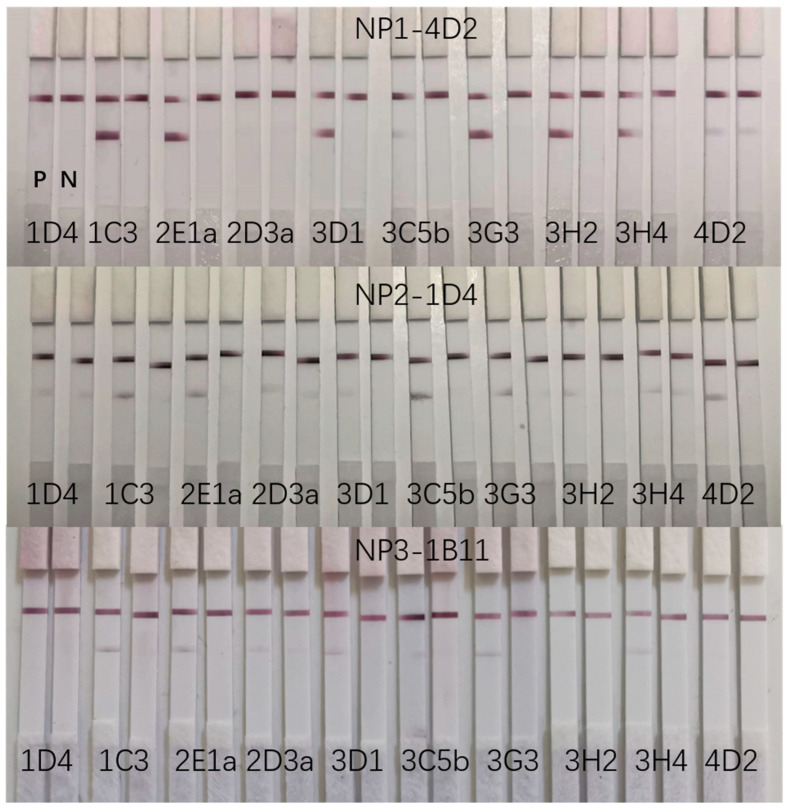
Screening of matched mAbs for 4D2, 1D4, and 1B11. P is 20 ng/mL recombinant N protein as the positive control. N is PBS as the negative control.

The antibodies targeting CoV-NP1, CoV-NP2, or CoV-NP3 could detect relatively high concentrations of the N protein when they were mutually paired in colloidal gold immunochromatography, most likely due to steric hindrance, which impeded the binding between the antigen and antibodies. In contrast, these CoV-NP1-, CoV-NP2-, and CoV-NP3-targeting antibodies could be well-matched by some of the N protein-targeting antibodies that we previously developed [6]. As shown in Figure 7, the CoV-NP1-targeting antibody 4D2 could be matched by several antibodies, with 1C3 displaying the most intense color. 1D4 and 1B11 could also be matched by some antibodies, but the color was relatively lighter when testing the N protein.

### 2.5. Sensitivity and Specificity of the Colloidal Gold Immunochromatographic Strips

**Figure 8:**
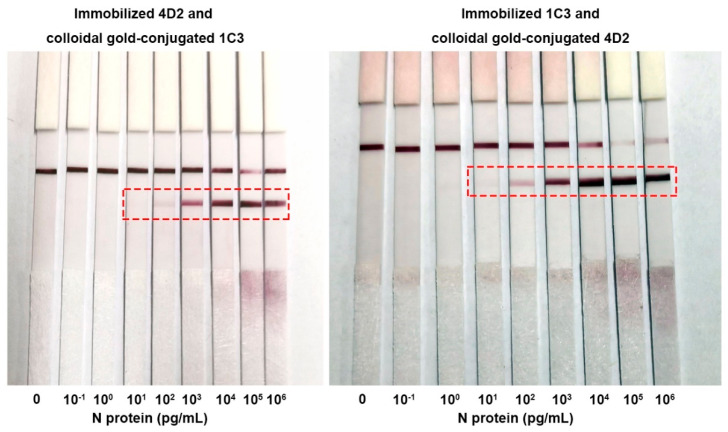
Sensitivity of using 4D2 and 1C3 in detecting the N protein of SARS-CoV-2 with colloidal gold immunochromatography. The concentrations of recombinant N protein were 0, 10^−1^, 10^0^, 10^1^, 10^2^, 10^3^, 10^4^, 10^5^, and 10^6^ pg/mL. Concentrations in the dotted boxes can be detected by the colloidal gold immunochromatographic strips.

We further investigated the detection sensitivity for the N protein when antibody 4D2 was paired with 1C3. As shown in Figure 8, immobilized 4D2 and colloidal gold-conjugated 1C3, and vice versa, showed a clear N protein concentration-dependent color gradient. The limits of detection (LOD) of both methods could reach 10 pg/mL.

**Figure 9:**
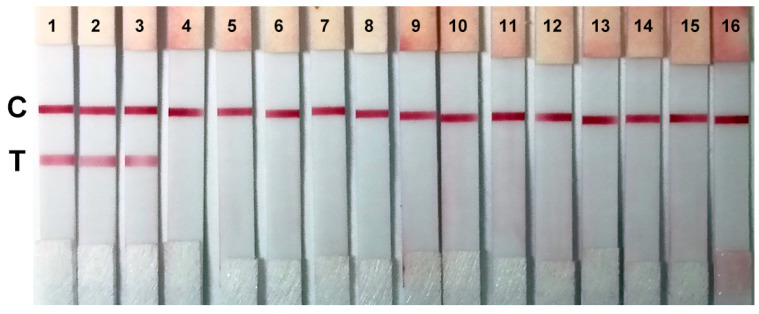
Cross-reaction verification. The colloidal gold immunochromatographic strips were used to test 1—SARS-CoV-2 B.1.1.7 (Alpha) N protein, 2—SARS-CoV-2 B.1.1.529 (Omicron) N protein, 3—SARS-CoV N protein, 4—MERS-CoV N protein, 5—HCoV-229E, 6—HCoV-OC43, 7—HCoV-NL63, 8—HCoV-HKU1, 9—influenza A (H1N1), 10—influenza B (Yamagata strain), 11—respiratory syncytial virus, 12—human parainfluenza viruses, 13—human bocavirus, 14—human metapneumovirus, 15—*Staphylococcus aureus*, 16—*Pseudomonas aeruginosa*. C is the control line. T is the test line.

We then characterized the specificity of the colloidal gold immunochromatographic strips composed of immobilized 1C3 and colloidal gold-conjugated 4D2 since these strips had relatively stronger signals (more intense test lines) at low concentrations of N protein (Figure 8). A series of respiratory pathogens were examined. As shown in Figure 9, in addition to the N proteins of SARS-CoV-2, the strip could only cross-react with the N protein of SARS-CoV due to its high similarity to the SARS-CoV-2 N protein [18]. This phenomenon is often reported in the literature [10]. The strips did not cross-react with other pathogens (Figure 9).

## 3. Discussion

Detecting antigens with immunoassays is recommended by the WHO, Centers for Disease Control and Prevention (CDC), and the European Center for Disease Prevention and Control (ECDC) for the diagnosis of COVID-19 [20]. The N protein is an important antigen that is often used in the detection of SARS-CoV-2 [10]. Although the N protein is relatively stable and conserved, mutations in N protein still occur [21], and disruptive mutations in the N protein may lead to a failure in detecting SARS-CoV-2 in large-scale population testing and transmission of the virus [22,23,24,25]. Therefore, the antibodies used for detecting the SARS-CoV-2 N protein should be reliable and not easily influenced by mutations. Antibodies targeting linear peptide antigens with conserved sequences are advantageous in this regard. On the one hand, mutations are less likely to occur in the conserved regions. On the other hand, even if mutations occur in the conserved epitope, it can efficiently verify whether the antibody can recognize the mutated antigen via synthesizing the mutated peptide. For unrecognizable mutated sequences, new antibodies can be rapidly prepared to avoid the potential failure of detection. Our study well-illustrated these advantages. Antibodies targeting the conserved peptide CoV-NP1 could effectively recognize both the natural antigen in the N protein and the mutated antigen.

Compared to nucleic acid tests, detecting the N protein with LFIAs is generally faster (usually within 30 min) [26]. However, the sensitivity of LFIAs (usually several hundreds of pg/mL) is relatively lower than nucleic acid tests, which is another challenge in detecting SARS-CoV-2. It has been reported that some commercially available rapid antigen detection kits are 10^2^–10^5^ times less sensitive than RT-PCR [27,28,29]. Great efforts have been made to enhance the sensitivity of LFIA for detecting SARS-CoV-2 [30]. For example, Ruantip et al. incorporated an enhancement pad to a conventional LFIA and achieved a 10-fold improvement in sensitivity (i.e., 0.5 ng/mL N protein) [31]. Kim et al. developed a non-powered preconcentrator for enriching the N protein for LFIA, which consequently improved the LOD by up to 10-fold [32]. Oh et al. applied gold nanoparticle clusters that were interconnected by the biotinylated antibody-streptavidin linkers to LFIA, which had an LOD of 38 pg/mL N protein, 23.8 and 5.9 times lower than LFIAs composed of 15 nm and 40 nm gold nanoparticles, respectively [33]. Hong et al. lowered the LOD of LFIA by two orders of magnitude to 0.24 pg/mL N protein by using SiO_2_@Au core core-satellite nanoparticles [34]. Peng et al. achieved a 1000-fold enhancement in LOD, from 10 ng/mL to 10 pg/mL N protein, of LFIA through copper deposition-induced signal amplification [35]. Ding et al. developed the upconversion nanoparticle-based immunochromatographic assay by replacing colloidal gold with upconversion nanoparticles. The LOD of this system was 3.59 pg/mL N protein, 100 times more sensitive than a commercially available colloidal gold LFIA [36]. With the help of the surface-enhanced Raman scattering (SERS) technique, Liu et al. greatly lowered the LOD of LFIA to 0.5 pg/mL for N protein by replacing traditional colloidal gold nanoparticles with Ag/black phosphorus nanosheets [37]. Our system, only through the rational selection of antigens and antibodies, achieved ultra-high sensitivity to the N protein, superior to the sensitivities of many analogous detection systems reported in the literature [6,31,38,39,40,41] and many commercially available detection products [42], manifesting the superiority of antibodies obtained by rational screening antigens with bioinformatical and structural biological means. We believe that by delicately designing the components of the LFIA, like the examples above-mentioned, we can further enhance the sensitivity of our LFIA system.

## 4. Materials and Methods

### 4.1. Screening of Peptides with Conserved Sequences as Antigens

The sequences of the N protein of SARS-CoV-2 variants were downloaded from the National Center for Biotechnology Information (NCBI). Sequence alignment was performed with the BioEdit software (version 7.0.9.0). The conserved sequences were obtained using our previously reported method [43].

The conformation of the N protein was predicted with Phyre^2^ [44]. The conserved sequences were labeled on the 3D structure of the N protein. Those located on the surface of the N protein, particularly located near the N terminus, were selected as the antigens for the following studies. The sequences of the selected peptides CoV-NP1, CoV-NP2, and CoV-NP3 are listed in Table 1. These peptides were synthesized and functionalized with cysteine at the N-terminus for conjugation with BSA or OVA.

### 4.2. Preparation of Monoclonal Antibodies

Six- to eight-week-old Balb/C mice were immunized intramuscularly on the leg with 50 μg BSA-conjugated peptide antigens, which were mixed with 10% Montanide Gel 01 PR as the adjuvant. A booster with the same dosage was given on day 14. Blood was collected from the tail on day 21 to evaluate the titer of antibodies using indirect ELISA with the immobilized N protein and HRP-conjugated goat anti-mouse IgG secondary antibody.

Mice NS1 myeloma cells were fused with activated B lymphocytes from the spleen to form hybridoma cells, which were cultured in the HAT medium. The positive single-cell clones that could produce N protein-targeting antibodies were screened using ELISA, and antibodies were purified by protein A affinity chromatography.

### 4.3. Evaluation of Binding Affinities

The antibodies were diluted with PBS and added to a 96-well plate that was pre-coated with 5 μg/mL recombinant N protein or 5 μg/mL OVA-conjugated peptide. After 1 h incubation and carrying out a washing procedure three times, the HRP-conjugated goat anti-mouse IgG was added and incubated for another hour followed by the washing procedure three times. Next, the absorption was measured at 490 nm in the presence of o-phenylenediamine dihydrochloride (OPD). The experiments were triplicated.

### 4.4. Investigation of the Inhibition of Binding between Antibody and N Protein by Different Peptides

The peptides were diluted with PBS and mixed with 100 ng/mL antibody. The mixture was added to a 96-well plate that was pre-coated with 5 μg/mL recombinant N protein and incubated for 1 h. After washing and incubation with the HRP-conjugated goat anti-mouse IgG, the absorption was measured at 490 nm in the presence of OPD. The experiments were triplicated.

### 4.5. Preparation of Colloidal Gold Immunochromatographic Strips

The N protein-specific antibody and goat anti-mouse IgG were immobilized at the test line and control line, respectively, on nitrocellulose membranes. The sample pads were prepared by treating glass fiber membranes with 1% BSA and 0.5% CaseiNBlock. The colloid gold-conjugated antibody that was prepared using our previously reported method [6] was deposited on conjugate pads. The colloidal gold immunochromatographic strips were prepared by mounting the nitrocellulose membrane, sample pad, conjugate pad, and absorbent pad onto the backing card and cutting it into 3 mm-width strips.

### 4.6. Screening of Matched Antibody Pairs

The N protein-specific antibodies that we previously developed [6] were conjugated with colloidal gold and deposited on conjugate pads of the colloidal gold immunochromatographic strips that were pre-coated with 4D2, 1D4, or 1B11 on the test lines. A matched antibody pair was determined when both test and control lines appeared in the presence of the N protein, while only the control line appeared in the absence of the N protein.

### 4.7. Evaluation of the Sensitivity and Specificity of Colloidal Gold Immunochromatographic Strips

To determine the sensitivity, 0–10^6^ pg/mL recombinant N protein was applied to the colloidal gold immunochromatographic strips and the test lines were examined after 10 min.

To determine the specificity, N proteins of SARS-CoV-2 B.1.1.7 (Alpha), SARS-CoV-2 B.1.1.529 (Omicron), SARS-CoV, and MERS-CoV, and different respiratory pathogens including HCoV-229E, HCoV-OC43, HCoV-NL63, HCoV-HKU1, influenza A (H1N1), influenza B (Yamagata strain), respiratory syncytial virus, human parainfluenza viruses, human bocavirus, human metapneumovirus, *Staphylococcus aureus*, and *Pseudomonas aeruginosa* were examined using the colloidal gold immunochromatographic strips.

## 5. Conclusions

In this study, we screened three linear peptides containing conserved sequences in the N protein of SARS-CoV-2 and successfully prepared mAbs targeting these three peptides. The epitope of CoV-NP1 was completely exposed to the surface of the N protein, as a result, antibodies targeting CoV-NP1 had the highest affinities for the N protein. The CoV-NP1-targeting antibody 4D2 could detect the N protein of SARS-CoV-2 with ultra-high sensitivity. Our study suggests that the bioinformatical analysis of conserved sequences combined with structural biological analysis provides a precise way to identify linear peptide antigens and the dominant epitopes, which is promising to solve the randomness and uncertainty of antibody discovery, thereby enhancing the sensitivity of LFIAs. This method could also potentially guide the rational design of peptide vaccines.

## Figures and Tables

**Table 1:** Sequences of peptides for preparing the antibodies.

Peptide	Sequence
CoV-NP1	PQNQRNAPRITFGGPSDST
CoV-NP2	SWFTALTQHGKEDLKFPRGQGVPINT
CoV-NP3	TRRIRGGDGKMKDLSP

## Data Availability

Data are available upon request.
